# Chemical Source Profiles and Toxicity Assessment of Urban Fugitive Dust PM_2.5_ in Guanzhong Plain, China

**DOI:** 10.3390/toxics11080676

**Published:** 2023-08-07

**Authors:** Ziyi Zhao, Jie Tian, Wenyan Zhang, Qian Zhang, Zhichun Wu, Yan Xing, Fei Li, Xinyu Song, Zhihua Li

**Affiliations:** 1 Key Laboratory of Northwest Resource, Environment and Ecology, MOE, Xi’an University of Architecture and Technology, Xi’an 710055, China; zhaoziyi@xauat.edu.cn (Z.Z.); wuzhichun@xauat.edu.cn (Z.W.); lizhihua@xauat.edu.cn (Z.L.); 2 Key Lab of Aerosol Chemistry & Physics, SKLLQG, Institute of Earth Environment, Chinese Academy of Sciences, Xi’an 710061, China; tianjie@ieecas.cn; 3 Zhongsheng Environmental Technology Development Company Limited, Shaanxi Environmental Protection Industry Group Company Limited, Xi’an 710065, China; wyzhang728@163.com; 4 Key Laboratory of Shaanxi Environmental Medium Trace Pollutants Monitoring and Early Warning, Shaanxi Environmental Monitoring Center, Xi’an 710054, China; xy18792833799@126.com (Y.X.); lifei0729@163.com (F.L.); m18789442264@163.com (X.S.); 5 Environmental Monitoring Station of Baqiao Branch, Xi’an Ecology of Environment Bureau, Xi’an 710038, China

**Keywords:** urban dust, source chemical composition, spatial variation, toxicity source apportionment, Guanzhong Plain

## Abstract

Urban fugitive dust is a significant contributor to atmospheric PM_2.5_ and a potential risk to humans. In 2019, both road dust and construction dust were collected from four cities, including Xi’an, Xianyang, Baoji, and Tongchuan, in Guanzhong Plain, China. Elements, water-soluble ions, and carbonaceous fractions were determined to establish the chemical source profile. High enrichment degrees of Se, Sc, Cl, and Zn in both road dust and construction dust indicated that the industrial system and energy consumption influenced Guanzhong Plain strongly. According to the coefficient of divergence, the two datasets within Xianyang and Tongchuan were similar. Combined with the chemical profile, road dust was affected by more stationary emission sources than construction dust in Xi’an, while biomass burning and vehicle exhaust contributed more to road dust than construction dust in Baoji. Moreover, the health risk of heavy metal was assessed, and corresponding influencing factors were identified. Road dust in all cities showed a non-negligible non-carcinogenic risk for children. Ingestion and inhalation were the main exposure pathways to which As and Co contributed the most, respectively. The land-use regression model revealed that the first-class road in a 100 m radius impacted all high-risk level metals, and the commercial building material and enterprises weakly influenced Co and Pb, respectively.

## 1. Introduction

Urban fugitive dust is a primary contributor to air particulate matter, especially in arid and semi-arid regions [1]. According to source apportionment results in previous studies, urban fugitive dust accounted for 11–61% as a significant source of air pollution [2,3,4,5]. Road dust and construction dust are the main types of urban fugitive dust. Road dust primarily originates from the Earth’s crust [6] and the friction between automobile tires and asphalt on paved roads [7,8]. Guttikunda [9] calculated that if a new car drives 180 km a week, 280 kg of PM_10_ will be resuspended each year. Construction dust is produced by construction activities surrounding building sites [10]. With the rapid economic development and urbanization in China, the length of roads and building areas expanded at a speed, and the urban fugitive dust issue came along with it and contributed partially to air pollution. Furthermore, toxic organic and inorganic pollutants, especially heavy metals (HM) in urban dust, present potential threats to the residents and ecosystems [11,12].

Many researchers conducted investigations into urban fugitive dust. Al, Fe, Si, Ca, and OC were abundant constituents in almost all resuspended fugitive dust samples (paved road dust, unpaved road dust, and soil dust) [13,14,15]. The high enrichment factor of Cu, Pb, Zn, Se, Sc, Cr, Cd, and so on indicated that these elements were strongly influenced by anthropogenic sources such as the metallurgical industry, vehicles, or coal combustion [15,16,17]. Sun et al. [10] investigated urban dust PM_2.5_ from 21 provincial capitals across China, and the results showed that urban dust in Northwestern China had the highest total carbon (TC) and elemental composition. HMs attached to urban dust have high persistence and biotoxicity, which could easily transfer into the human body by inhalation, ingestion, or dermal contact, then affect the function of kidneys, lungs, or other organs and lead to the skeletal, cardiovascular, and cerebrovascular diseases [18,19]. Previous studies determined the concentration of heavy metals in road dust from Northwestern Chinese cities and estimated the health risk of heavy metals [20,21,22,23]; it turns out that Cr, As, and Pb were the main threatening metals to local residents. Wang et al. [24] found that the non-carcinogenic risk of Pb in commercial districts in Nanjing for children was higher than the safety threshold. Shahab et al. [25] revealed that the carcinogenic risk via ingestion (CR_ing_) illustrated significant risks to children from Cr, As, and Ni in urban, industrial, and tourist areas. Although source apportionment was conducted for HM in urban dust in the aforementioned studies, the scale was not specific enough. The effects and contributions of the intraurban factors were still lacking. Land-use regression (LUR) is a robust technique that utilizes a simple linear regression to predict the concentrations of pollutants at a given site based on the surrounding physical environment, land use, and traffic characteristics [26]. It has the advantages of low data requirements, complete consideration factors, high simulation accuracy, high spatial resolution, and a wide application range [27]. The LUR model was used to predict the pollutants in atmospheric PM_2.5_ [28,29]; subsequently, the LUR method was increasingly used in explaining and predicting the heavy metals in urban dust or soil [26,27,30,31]. Thus, it is crucial to determine what characteristics of the urban form act as driving factors of heavy metal pollution in urban dust to obtain a better measurement that limits the exposure to heavy metals [30].

Guanzhong Plain is a semi-arid region located in the middle of Shaanxi Province, China. It is also an important part of Fenwei Plain—designed as one of the three key regions for the “Blue Sky Protection Campaign” in China. According to previous studies, air pollution in Guanzhong Plain is serious. During an extreme pollution event in January 2015 in Xi’an, the average PM_2.5_ concentration was ~250 μg/m^3^ with a peak of ~700 μg/m^3^ [32]. The urban fugitive dust in the four cities mentioned above contributed 12.46%, 9.36%, 13.69%, and 9.48% to aerosol PM_2.5_, respectively, from November 2018 to January 2019 [33]. Few studies have a simultaneous investigation of the urban dust from the Guanzhong city cluster; it is necessary to conduct a comprehensive field study to obtain the chemical profiles and health risk assessment of urban dust PM_2.5_ in the Guanzhong Plain. Four aims were set in this study: (1) build the source profile of urban dust PM_2.5_ in Guanzhong Plain by determining the concentration of elemental composition, water-soluble ions, and carbonaceous fractions; (2) according to the divergence of chemical characteristics among cities, identify the main influence sources in each city quantitively; (3) calculate the non-carcinogenic and carcinogenic risks of heavy metals for both children and adults; (4) utilize the Land-use Regression model to figure out the function of heavy metal with high-level risk, analyzing the contribution of intraurban factors.

## 2. Materials and Methods

### 2.1. Study Area

Xi’an (XA), Xianyang (XY), Baoji (BJ), and Tongchuan (TCH) were chosen to be the study areas. The brief information on selected cities is listed in Table 1.

XA is the capital of Shaanxi Province and is the largest city in Northwestern China; all the samples in XA were collected within the Third Ring Road. XY is located northwest of XA; the downtowns of the two cities are only about 25 km apart. BJ is the second largest city in Shaanxi province, located in the western Guanzhong Plain, bordering Gansu province. TCH is a traditional industrial city in the north of Guanzhong Plain; after 2000, a new district was built as the new political, economic, and cultural center located about 30 km southwest of the old downtown.

### 2.2. Sampling Collection

The sampling collection period started in July 2019 and finished in November 2019; a total of 30 road dust samples and 24 construction samples from four cities were collected. Road dust (RD) was collected from the curb of the main road, and Construction dust (CD) was collected from the ground in construction sites or the iron plates at the gate for trucks to roll. We used the long-handled brush to collect dust, then swept dust into a clean plastic dustpan, stored in self-sealed polyethylene bags, and labeled them after sieving using a 1.0 mm mesh to remove the small stones, branches, and other debris. The detailed location of the sampling sites is shown in Figure S1.

### 2.3. Resuspension

**Figure 1:**
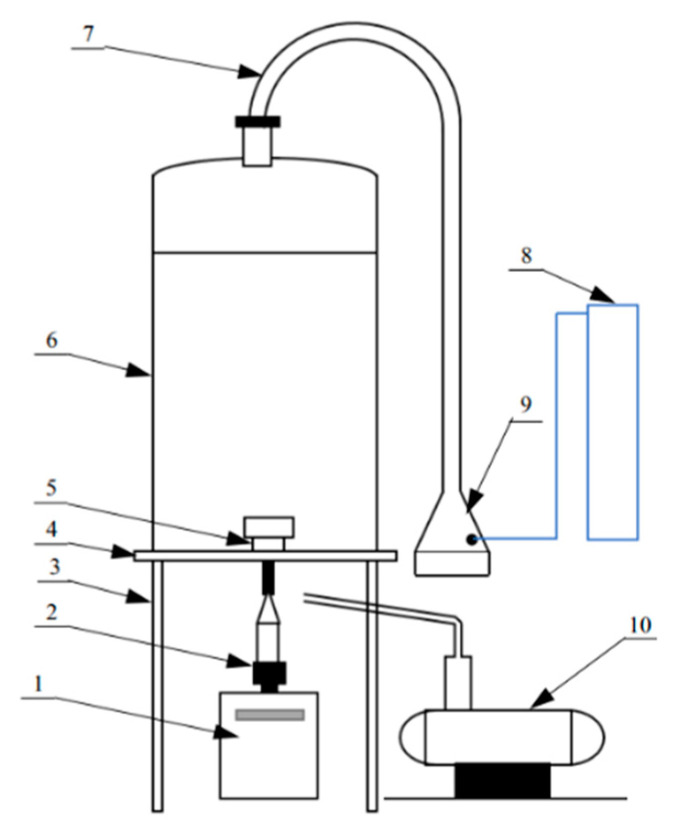
Sketch map of dust resuspension installation and sampling equipment. (1) mini-vol sampler; (2) PM_2.5_ cutter; (3) stand; (4) resuspension platform; (5) joint; (6) resuspension chamber; (7) diffuser tube; (8) nitrogen tank; (9) sample plate; (10) vacuum pump.

A self-made resuspension installation designed by the Institute of Earth Environment, Chinese Academy of Sciences (IEE-CAS) was used to separate PM_2.5_ samples from the raw dust; the structure of the instrument is shown in Figure 1. Three spoons of raw dust, approximately 0.5 g, was placed in the sample plate at the end of the diffuser tube; subsequently, the resuspension system was vacuumized for 5 min by a pump and then impacted high-pressure nitrogen once per minute into the sample plate to resuspend raw dust and to simulate the deposition process in the resuspension chamber. Mini-volume sampler (Airmetrics, Springfield, OR, USA) was employed to collect the PM_2.5_ samples at a flow rate of 5 L/min. The resuspension installation was modified and developed by Chow et al. [34]. There was a capture efficiency test from a similarly developed resuspension chamber. The cut size of the PM_2.5_ impactor was 2.43 μm; the size range was 1.78–3.21 μm; the corresponding efficiency ranged from 14.7% to 92% [35]. An available PM_2.5_ sample on a 47 mm diameter quartz microfiber filter (Whatman Limited, Maidstone, UK) should be collected for at least 6 min. Each filter was preheated to 800 °C for 3 h to remove organic compounds on the filter [1].

### 2.4. Chemical Analysis

#### 2.4.1. Filter Weighing

Sartorius ME 5-F electronic microbalance with a sensitivity of ±1 μg (Sartorius, Gottingen, Germany) was used to weigh filters; each filter should be placed in an environmental chamber to equilibrate for 24 h at a temperature between 20 and 23 °C and relative humidity between 35% and 45% before weighing [36]. Each filter should be weighed at least two times both before and after use. The net mass of the PM_2.5_ sample is the difference between the average blank weight and the average post-sampling weight.

#### 2.4.2. Elemental Analysis

For elemental analysis, each quartz filter was pressed into a 32 mm diameter pellet under 30 N pressure for 20 s, then the concentrations of 19 elements (Cl, K, Ca, Ti, Sc, V, Mn, Fe, Co, Ni, Cu, Br, As, Se, Sr, Ba, Zn, Ga, and Pb) were directly measured by Energy Dispersive X-Ray Fluorescence (ED-XRF) spectrometry (Epsilon 5 ED-XRF, PANalytical B. V., Almelo, the Netherlands). Each filter was analyzed for 32 min at 30 °C to obtain X-ray spectrum counts versus photon energy, individual peak energies matching specific elements, and peak areas corresponding to elemental concentrations. Detail description of XRF analysis was given by Xu et al. [37]. The ED-XRF spectrometer was calibrated with thin-film standards obtained from Micro Matter Co. (Arlington, WA, USA).

#### 2.4.3. Water-Soluble Inorganic Ion Analysis

A quarter of the filter was used to determine major cations (Na^+^, NH4 ^+^, K^+^, Mg^2+^, and Ca^2+^) and anions (SO_4_^2−^, NO^3−^, Cl^−^, F^−^, and NH_4_^+^). Filter was cut up into a 15 mL centrifuge tube with 10 mL ultrapure water (resistivity of 18 MΩ·cm) added, then shaken by ultrasonic instrument (KQ5200DE, Kunshan ultrasonic instrument) for 60 min and then by mechanical shaker for 60 min; the ions were extracted completely. The extracting solution was filtered into the sample bottles and determined by ion chromatography (IC, Dionex Integrion HPIC, Dionex Corp, Sunnyvale, CA, USA). A CS12A column (Dionex Company) with 20 mM MSA eluent and an AS14 column (Dionex Company) with 3.5 mM: 1 mM Na_2_CO_3_ and NaHCO_3_ mixture as the eluent was used for determining the concentration of cations and anions, respectively.

#### 2.4.4. Carbonaceous Fraction Analysis

A 0.5 cm^2^ punch was cut from a 47 mm quartz filter to be determined eight carbonaceous fractions by DRI Model 2001 Thermal and Optical Carbon Analyzer (Atmoslytic Incorporated, Calabasas, CA, USA) following the Interagency Monitoring to Protect Visual Environments (IMPROVE) protocol [38,39]. OC1, OC2, OC3, OC4 were volatilized and measured at 120 °C, 250 °C, 450 °C, and 550 °C in a pure helium atmosphere, respectively. EC1, EC2, and EC3 were combusted to CO_2_ and measured at 550 °C, 700 °C, and 800 °C in a 2% oxygen/98% helium atmosphere, respectively. When heated in an inert helium atmosphere, some OC pyrolyzed to light-absorbing EC; then oxygen was added; this pyrolysis char combusts, along with the original EC collected on the filter’s surface, the pyrolyzed OC named OP. The IMPROVE protocol defines OC as OC1 + OC2 + OC3 + OC4 + OP and EC as EC1 + EC2 + EC3 − OP.

### 2.5. Data Processing

#### 2.5.1. Enrichment Factor

The enrichment factor is calculated to further investigate the influence of anthropogenic sources on elements in fugitive dust. In this study, Fe was used as the reference element. The concentration list of topsoil in Xi’an’s report by Cheng et al. [40] was used as the geochemical background concentration for the four aimed cities, and the EFs were calculated by the following equation:

(1) $${EF}_{x}={\left(X/Y\right)}_{sample}/{\left(X/Y\right)}_{crust}$$

where *EF_x_* means *EF* of element *X* in dust samples; (*X*/*Fe*)*_sample_* means the proportions of elements X and Fe in samples, and (*X*/*Fe*)*_crust_* means the proportions of elements *X* and Fe in Earth’s upper continental crust (UCC) as well as the topsoil in this study. Generally, if the *EF* of an element was <5, the element was deemed to have originated from UCC, and if the *EF* was >5, the element was contributed by anthropogenic sources [41].

#### 2.5.2. Coefficient of Divergence

Coefficient of divergence (*COD*), a self-normalizing parameter, is used to accurately describe the inter- and intra-regional similarities in the two types of fugitive dust profiles. The *COD* was calculated using the following equation:

(2) $$COD=\sqrt{\frac{1}{P}\sum_{i=1}^{P}{\left(\frac{x_{ij}-x_{ik}}{x_{ij}+x_{ik}}\right)}^{2}}$$

where *x_ij_* represents the average concentration for a chemical component *i* at site *j*; *j* and *k* represent two sampling sites, and *p* is the number of chemical components. If the two sampling sites are similar, the CD approaches zero. If the two sampling sites are very different, the CD approaches one [42].

#### 2.5.3. Human Health Risk Assessment of Heavy Metal

The non-carcinogenic and carcinogenic health risks ascribed to the intake of heavy metals in road dust were assessed by the model developed by the US Environmental Protection Agency [43]. The average daily dose (*ADD*) of three exposure pathways (ingestion, inhalation, and dermal contact) of every heavy metal is assessed as follows:

(3) $${ADD}_{ing}=C\times \frac{ingR\times EF\times ED\times CF}{BW\times AT}$$

(4) $${ADD}_{inh}=C\times \frac{inhR\times EF\times ED}{PEF\times BW\times AT}$$

(5) $${ADD}_{ing}=C\times \frac{SL\times SA\times ABS\times EF\times ED\times CF}{BW\times AT}$$

where *C* is the concentration of heavy metal in urban dust; *ingR* is the ingestion rate; *inhR* is the inhalation rate; *EF* is the exposure frequency; *ED* is the exposure duration; *SA* is the exposed skin area. In this study, *SL* is the skin adherence factor, *ABS* is the dermal absorption factor (unitless). *PEF* is the particle emission factor, *BW* is the average body weight, and *AT* is the average time. For non-carcinogens, *AT* (days) = *ED* × 365; for carcinogens, *AT* (days) = 70 × 365 = 25,550. The values of all parameters [43,44,45] are listed in Table S1.

The Hazard index (*HI*) is used to estimate the non-carcinogenic risk of heavy metals and is the sum of the Hazard quotient (*HQ*) of three exposure pathways. Excessive lifetime cancer risk (*CR*) attributed to specific heavy metals was calculated by multiplying *ADD* by slope factor (*SF*). The equations of *HQ*, *HI*, and *CR* calculation were defined as follows:

(6) $${HQ}_{i}=\frac{{ADD}_{i}}{{{(R}_{f}D)}_{i}}$$

(7) $$HI=\sum_{i=1}^{n}{HQ}_{i}$$

(8) $${CR}_{i}={ADD}_{i}\times {SF}_{i}$$

(9) $$TCR=\sum_{i=1}^{n}{CR}_{i}$$

where *ADD_i_* and *(RfD)_i_* are doses attributed to pathway *i* and corresponding reference dose of each heavy metal; *n* is the number of exposure pathways; *SF_i_* is the carcinogenic slope factor. The values of *R_f_D* and *SF* [46,47] are presented in Table S2.

#### 2.5.4. Establishment of the LUR Models

To identify the main intraurban land-use factors affecting heavy metal and corresponding health risk distributions, maps in a GIS were used to generate 10 spatial variables (Table S3). The land-use data were obtained from the Chinese National Platform for Common Geospatial Information Services.

Pearson correlation analysis was carried out for each variable and the contents of heavy metals by SPSS 27.0. The influencing variables with a significant correlation to aimed dependent variables were retained. Using these influencing variables to calculate the linear regression equation, the form is as follows:

(10) $$y=b_{0}+b_{1}x_{1}+b_{2}x_{2}+\ldots b_{n}x_{n}$$

where *y* is the dependent variable which represents the health risk of the heavy metal in urban dust; *b*_0_, *b*_1_…, *b_n_* are constants; *x*_1_, *x*_2_…, *x_n_* are the influence (independent) variables which were significantly related to dependent variables. To ensure the low risk of multicollinearity that could bias the coefficient, the variance inflation factor (VIF) of each influencing variable must be below 5 [30].

## 3. Results and Discussion

### 3.1. Characteristics Fugitive Dust-Related Elements

The elemental chemical profiles of two types of urban fugitive dust from four selected cities are listed in Supplementary Table S4. The average proportion of total elements (19 species) in total RD PM_2.5_ from four cities followed the sequence of XA (43.75%), XY (35.88%), BJ (36.97%), and TCH (30.37%), respectively. The most abundant elements which were above 1% included Ca, Fe, and K. Ca was the absolutely dominant element ranging from 22.59 to 33.96% in RD, which was comparable to that in the cement plant in Northwestern China (25.68%) [48], but was higher than in many other regions or cities in China (4–20%) [10,17,49,50]. Ca in urban fugitive dust is not only from crustal soil but also from the cement industry or construction activity [51]. The rest two abundant elements, Fe (3.79–5.15%) and K (2.69–4.51%), also mainly originate from the Earth’s UCC. The average proportions of total elements in CD PM_2.5_ from four cities followed the sequence of XY (36.45%), TCH (32.75%), XA (32.25%), BJ (30.14%), respectively, which were slightly lower than those of RD. There was a similar pattern of elements in RD of each city; Ca was still the most abundant element. In contrast to RD, the maximum average concentration of many elements in CD PM_2.5_, including Ca, Cl, Sc, and Cu, turned to be XY instead of XA and BJ instead of TCH as minimum amounts.

**Figure 2:**
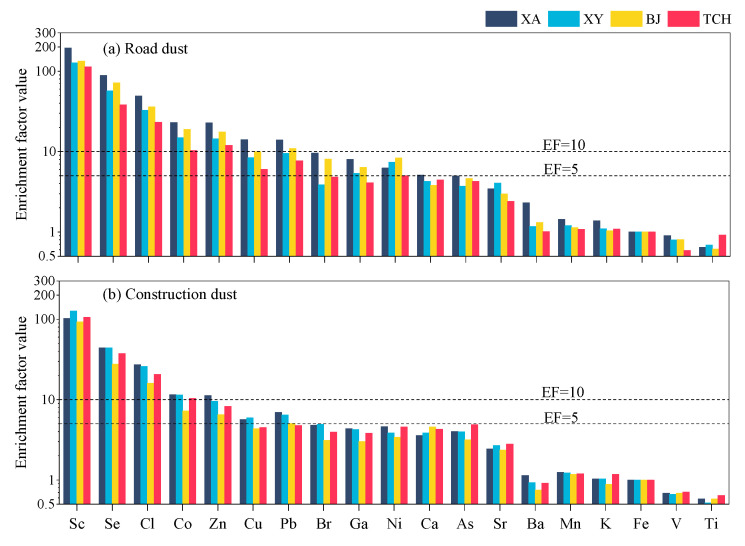
Enrichment factor of (**a**) RD and (**b**) CD in four selected cities.

The Enrichment factor (EF) was used to conduct an assessment of the influence of anthropogenic sources on the elements. Figure 2 shows the enrichment factor value of each measured element in two types of dust from four cities. High EF values (>10) of Se, Sc, Cl, and Zn in RD followed the sequence of XA > BJ > XY > TCH, while XA > XY > TCH > BJ in CD were all over 5, indicating the anthropogenic influences. Sc was strongly enriched in RD and CD PM_2.5_ in all cities, with an EF value over 90. Combined with the fields of application and character of industry in Guanzhong Plain, the national defense industry, aerospace and aviation industry, and steel industry should be the main sources [52,53,54,55,56,57]. For Se, it might enrich easily in the surface soil because it could form a stable complex by reacting with humic acid in the soil [58]. Meanwhile, the abundance of Se in the Chinese Loess Plateau was closely related to the coal combustion and biomass burning emissions [15]. High Cl EF values were mainly attributed to the contributions of coal combustion, biomass burning, and wind-eroded soil dust in inland region [1,59]. Zn could also come from the fly ash of the metallurgical industry [48,60]. Combining with Se, Sc, and Cl, the enrichment of these four elements mutually indicated the strong influence of the steel industry and coal combustion on the dust in Guanzhong Plain. Energy consumption of industrial enterprises in four selected cities in 2019 followed the sequence of XA (6.35 million) > XY (5.76 million) > BJ (5.73 million) > TCH (3.32 million) (unit: tons of standard coal) [61]; it was similar to the order of cities by these elements. In addition, the EF value of Cu and Pb in both RD and CD were in the range of 4–14, reflecting mixed origins from anthropogenic and natural sources. Cu could be discharged from the abrasion of the brake lining and tire of the motor vehicle [16]. Though there was a restraining order on the use of leaded gasoline in China since 2000, both the wear of lead on the tire and the residual deposit of leaded gasoline combustion that contained Pb were the main reasons for high Pb EF values in urban fugitive dust [1]. These two elements mutually indicated the motor vehicle source, which could easier become enriched in RD than in CD. The registered vehicles of the four cities followed the decreasing order of XY (3.43 million) > XY (0.46 million) > BJ (0.39 million) > TCH (0.10 million) in 2019 [61]; it was basically consistent with the order of cities by these elements.

### 3.2. Levels of Water-Soluble Inorganic Ions (WSI) and Carbonaceous Matter

**Figure 3:**
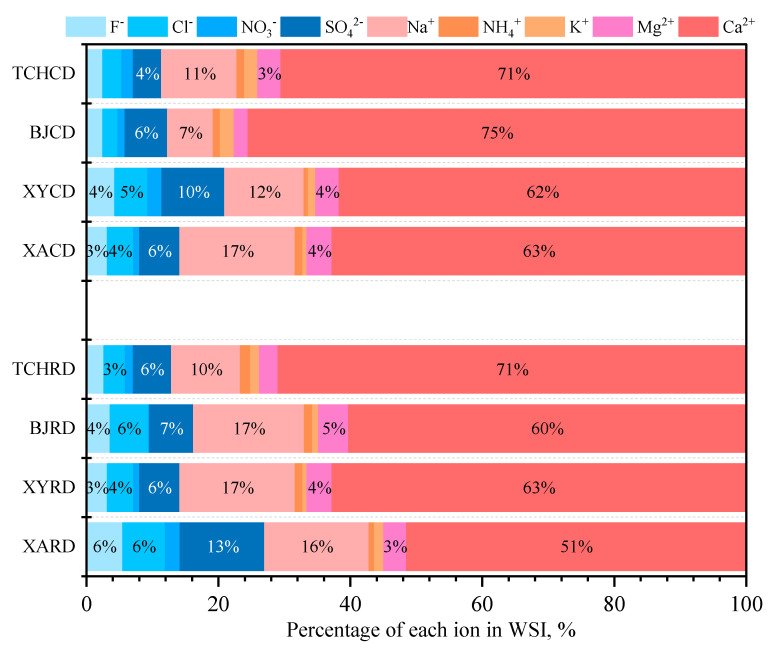
Water−soluble inorganic ions proportion in selected cities.

In Table S5, the total measured 9 WSIs occupied 28.53% (XA), 31.30% (XY), 25.75% (BJ), and 18.66% (TCH) in RD PM_2.5_, respectively, which were much higher than those in other cities or regions such as Zhengzhou (11.2%) [62], Tianjin (11.65%) [63], Shijiazhuang (22.11%) [64], and Pearl River Delta city cluster (14.17%) [65]. However, these fractions were comparable to the level of clean urban aerosol (~30%) [66] and were far smaller than haze day (~50%) [67]. Ca^2+^ was still the largest proportion in total WSIs in accord with element composition, proximity to the level of RD in the Northeastern China region [1]. The dramatically high Ca^2+^ of total WSIs in TCH (71%) showed the severe contamination by calcium-rich building material or construction dust that occurred in this urban area (Figure 3). The second largest cation was Na^+^ in RD PM_2.5_; it might be attributed to the use of road salt, an economical and effective deicer for road maintenance in wintertime [68,69]. SO_4_^2−^ emitted from numerous stationary source emissions was the most abundant anion in RD PM_2.5_ in XA (~13%), which was nearly twice as in other cities (~6%). For CD PM_2.5_ samples, the total WSIs proportion accounted for 18.22% in XA, 28.44% in XY, 15.10% in BJ, and 24.98% in TCH, respectively, which were slightly lower than those levels of RD but still much higher than in other regions. For example, Sun et al. [10] collected and determined 21 capital cities in China; the total WSIs varied from 4.2% to 16.4%. These phenomena stressed that the WSIs had become key pollutants in CD in recent years. Compared with RD, the WSI in CD of XA and BJ decreased sharply, while TCH is the only city that owned a higher WSI in CD than in RD. Similar to RD, Ca^2+^ was also the highest fraction of total WSI; the value increased in XA and BJ, and the WSI value decreased, inferring that the construction dust in XA and BJ was less influenced by other sources.

**Figure 4:**
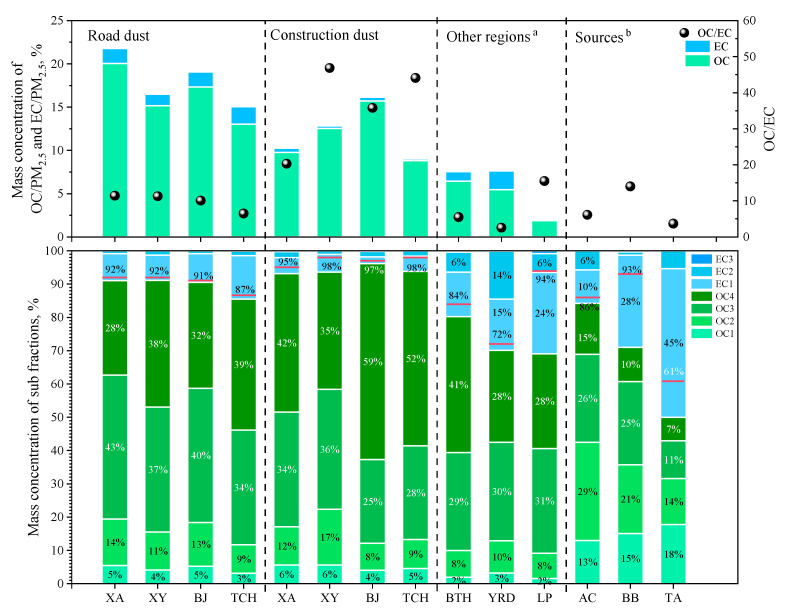
Carbonaceous fractions of urban dust in Guanzhong Plain and other regions and emission dust in sources. a: BTH: road dust in Beijing–Tianjin–Hebei Region, consisting of 5 cities; YRD: road dust in Yangtze River Delta Region, consisting of Shanghai and Nanjing [10]; LP: soil dust of suburb area in Chinese Loess Plateau [15]. b: AC: Anthracite coal; BB: Biomass burning [70]; TA: aerosol in tunnel [71]. Red line: EC1 fraction under red line is OP; EC1 fraction above red line is EC1-OP; the fraction under red line is OC.

The carbonaceous fraction profiles of two types of urban fugitive dust from four selected cities were listed in Table S7; the TC/PM_2.5_, OC/EC, and the relative concentration of individual sub-fractions were performed in Figure 4, including RD and CD in four selected cities and other regions, the sources of previous studies. The TC/PM_2.5_ in RD followed a descending order of XA (21.79%) > BJ (19.05%) > XY (16.46%) > TCH (15.05%), respectively; these fractions were higher than those fractions in soil dust PM_2.5_ in Chinese Loess Plateau (LP) (2.00%), RD in Beijing–Tianjin–Heibei Region (BTH) (7.60%) and RD in Yangtze River Delta Region (YRD) (7.62%), implying that human activities exhaust amount of carbonaceous fractions to Guanzhong urban area. OC/EC in RD was similar in four cities. Sub-fraction of OC, OC1, OC2, OC3, OC4, and OP had similar patterns in four cities with RD TC. Moreover, the EC1-OP fraction in RD TC was the highest in TCH TC, reflecting the more relative influences of bituminous coal combustion and vehicle emissions [70,71]. Guanzhong Plain RD TC had higher OC3 and lower EC1 and EC2 fractions in contrast to those in BTH and YRD regions, probably inferring that Guanzhong Plain suffered more from cooking oil fumes and less from motor vehicle emissions [72]. For CD, the TC/PM_2.5_ in CD followed the sequence of BJ (16.14%) > XY (12.81%) > XA (10.24%) > TCH (9.02%), with a lower average of 12.05% than RD; an explanation for that was that road dust accepted more traffic-related anthropogenic emission including larger traffic flow and cuisine exhaust. OC/EC in CD varied from 20.3 to 46.8, which were well above those in RD due to the extremely high OC level. OC4 fraction in CD was 38% higher than in RD, which might be attributed to the effect of the coal combustion [72]. OP fraction took up a higher proportion in CD (2.5 times higher than in RD) compared to the biomass burning (BB) column in Figure 4, implying that building material was more affected by this source [70,73,74].

### 3.3. Comparability of Source Profiles in Fugitive Dust

Coefficient of divergence (CoD) is used to accurately describe the inter- and intra-regional similarities in two types of fugitive dust profiles [10]. Considering the difference between RD and CD PM_2.5_ compositions, the CoD values between RD and CD in a city were also calculated in decreasing order: BJ (0.43) > XA (0.32) > TCH (0.20) > XY (0.19). Two datasets within XY and TCH both showed strong similarity, while the CoD for XA and BJ revealed the non-neglected divergence between RD and CD. The highest SO_4_^2−^/Ca^2+^ value was observed in XA RD than that in other cities (Table S6), proving that XA RD was influenced by intense stationary emission sources in addition to the vehicle emissions [10]. A higher ratio of K^+^/K and NO_3_^−^/SO_4_^2−^ was observed in BJ CD than those observed in BJ RD, suggesting the enhanced influences of biomass burning and vehicle emissions [75,76]. As shown in Table 2, for spatial distribution, the values of RD CoD among these four cities were higher than the threshold (0.2) and could even be up to 0.39 between XA and TCH, indicating that the chemical profiles of RD PM_2.5_ were dissimilar across the Guanzhong urban areas. For instance, the elements with high enrichment degree owned almost twice the EF value in XA as those in TCH, proving that XA RD might suffer more coal combustion and industrial emission, while high abundances of relative Ca^2+^ and EC1-OP fraction indicated that more construction activities and traffic influence existed in TCH. However, in CD, the CoD values between XA and XY, XA and TCH were below 0.2, implying similar sources. It could be explained by the fact that the building material was similar or was from the same place (such as a cement plant) and by the lower human activity effect. In comparison, the CD in BJ expressed divergence between the cities (CoD = 0.22–0.34). Meanwhile, the lowest NO_3_^−^/SO_4_^2−^ ratio (0.17) was observed in BJ CD, which was close to that in the fly ash of iron smelt plant source (0.1) [48], probably pointing out that the key contributions of stationary source discharge influenced by the BJ CD were different from other cities.

### 3.4. Source Apportionment of Health Risk in Fugitive Dust

#### 3.4.1. Health Risk Assessment

To evaluate the health risk of fugitive dust over urban areas, both the non-carcinogenic risk and carcinogenic risk of specified heavy metal elements of RD and CD PM_2.5_ in four selected Guanzhong cities were calculated in Table 3. For children, the hazardous index (HI) values of total heavy metals (THM) for RD and CD all exceeded the USEPA’s guideline value of 1, but for adults, there were potential adverse health effects only in XA RD and BJ RD. These phenomena manifested that children were more vulnerable to non-carcinogenic risks from urban dust. The non-carcinogenic risks of THM among cities in RD and CD follow the orders of XA > BJ > XY > TCH and XY > XA > TCH > BJ, respectively, which were consistent with the concentration order of THM. Discussing the hazardous quotient (HQ) of THM, HQ_ing_ and HQ_inh_ for RD and CD had an order of magnitude higher than HQ_dermal_, indicating that direct pathways (ingestion and inhalation) are the main exposure routes and are more dangerous than the indirect pathway (dermal contact); a similar finding was reported by former studies [77,78].

For the individual heavy metal risk, the HI value for children in RD and CD had the decreasing rank of As, Co, Pb, Ni, Cu, and Zn, which were completely different from the concentration rank of Zn, Pb, Cu, Co, Ni, and As. The HI values of Co, As, and Pb in RD were at an alarming rate, which exceeded 1 while lying at acceptable levels in CD. Regarding individual HQ values for specified metal elements, the ingestion exposure of Ni, Cu, Pb, and Zn to RD and CD exhibited the highest risk, followed by inhalation and dermal contact. Especially, the HQ of Pb through ingestion had a magnitude two–three times higher than the other two pathways. However, the element As in RD and CD reached the highest non-carcinogenic risk with the lowest concentration, which had a slightly different risk sequence of the pathway of HQ_ing_ > HQ_dermal_ > HQ_inh_ from other metal elements. The potential non-cancer risks attributed to exposure to As and Pb are the incidence of diseases in the respiratory tract, gastrointestinal tract, kidney, liver, skin, cardiovascular, nervous, and hematopoietic systems [46]. The inhalation exposure of Co for children was the key pathway and was higher than the acceptable level of 1 in RD, the magnitude of which was approximately two–three times higher than that for other elements. For adults, the HI value of every individual heavy metal was below the threshold both in RD and CD. These results confirmed that non-carcinogenic risk caused by RD to local children in Guanzhong Plain should be of concern, especially attributed to children’s repetitive hand-to-mouth touching behavior [79]. In addition, the rapid growth capability of children may intensify the adverse effects of heavy metals in their body [80].

Further, carcinogenic risks ascribed to Co, Ni, As, and Pb through the inhalation pathway were estimated. The total carcinogenic risk (TCR) of RD and CD PM_2.5_ in four Guanzhong cities were all in the range of 10^−6^~10^−4^, which was an internationally accepted precautious criterion [81], revealing the non-negligible but low level and acceptable cancer risk of THM in fugitive dust [46,82,83].

#### 3.4.2. Source-Attributed Health Risk Using LUR Regressions for Health Risk Assessment

To investigate the high non-carcinogenic risks and how they are influenced by intraurban factors, land use regression (LUR), models of HI (THM), HI (Co), and HI (Pb) for children were estimated in Table 4. The explained variation ranged from 14.4% to 23.0%, indicating a relatively low explanation degree; it could interpret the absence of other intraurban factors, such as population density and specific traffic load. The contributing factors in this study were mainly about traffic. It is noticed that a transport station in a 100 m buffer radius (TransStation100) was used in all equations, which included bus stops, metro stations, and train stations, implying that intensive traffic activities and crowded areas contributed to non-carcinogenic risk in general. Previous studies also reported that motorized traffic was an important source of intraurban spatial variation [28]. For HI (Co) model, the standardized coefficient (β) of the first-class road in buffer 100 m (1stRoad100) and TransStation (1500) (Table S8) were comparable, and about half of TransStation (100), slightly higher than the number of restaurants in buffer 300 m (Restaruant300). Co exists in alloy and stainless steel of automobile parts [84]. Cooking did not exhaust Co, but the restaurants are usually located in busy commercial areas related to commercial complexes. The erosion of building decorative elements and lamps could lead to the accumulation of Co [85,86]. A number of enterprises in buffer 1500 m (Enterprise1500) were affected weakly by HI (Pb) with β of 0.081. Besides traffic influence, Pb was also emitted by heavy pollution enterprises such as the pharmaceuticals [26]. Hence, controls of emissions from urban vehicles, the use of building materials, and pharmaceutical companies can help curb the health risks of fugitive dust in Guanzhong urban areas. In addition, more anthropogenic factors, including corporate emission inventories, will be considered in our future LUR model, which can play an important role in the comprehensive assessment of the health risks of important chemical compositions in fugitive dust.

## 4. Conclusions

The chemical source profile of urban dust, which includes road dust (RD) and construction dust (CD) in Guanzhong Plain, presents elements, water-soluble ions, and carbonaceous fractions. Elements composed the highest proportion (36.74% in RD, 32.90% in CD). Ca dominated in every dust sample. High enrichment factor values of Se, Sc, Cl, Zn, Cu, Pb, and Zn in RD almost all followed the sequence of Xi’an (XA) > Xianyang (XY) > Tongchuan (TCH) > Baoji (BJ), indicating the intensive influence of steel industry, coal combustion, and motor vehicle. Compared to other regions and cities, water-soluble inorganic ions (26.06% in RD, 21.69% in CD) and total carbon (18.09% in RD, 12.05% in CD) were much higher in Guanzhong Plain. According to the coefficient of divergence values, RD in XA and TCH differed significantly. Combined with the chemical profile, XA RD might suffer more coal combustion and industrial emission while more construction activities and traffic influence exist in TCH. Non-carcinogenic risk of total heavy metal for children was at an alarming rate in all cities. Heavy metals in dust caused damage to children mainly posed through ingestion (Ni, Cu, Pb, and Zn) and inhalation (Co) routes. There was no carcinogenic risk of urban dust in Guanzhong Plain. The land-use regression models for metals with high hazardous index values were at a low explanation degree (14.4–23.0%). TransStation100 includes a bus stop, metro station, and train station in a radius of 100 m, affecting all dependent variables significantly, implying intensive traffic activities, and crowded areas contributed to non-carcinogenic risk in general. Besides traffic-related factors, restaurants and enterprises contributed to non-carcinogenic risks weakly. The research results of this study are of great value because of the source analysis of local ambient air particulate matters and their toxicity in Guanzhong Plain and provide a scientific basis for formulating emission control policies. However, more toxicity factors such as polycyclic aromatic hydrocarbons or reactive oxygen species in urban fugitive dust should be proposed in future studies to evaluate public health more sufficiently.

## Figures and Tables

**Table 1:** Brief information on selected cities.

City	Latitude	Longitude	Sample Number	Industrial System	Population (Million, 2019)
XA	34°20′33.85″ N	108°56’22.69″ E	23	National defense industry, equipment-manufacturing industry, and high-tech industry.	10.2
Y	34°19′47.74″ N	108°42′32.59″ E	11	Chemical industry, equipment-manufacturing industry, food industry, and building materials industry.	4.36
BJ	34°21′47.42″ N	107°14′15.89″ E	10	Iron and steel mill, chemical industry, ceramics, cement plant, paints, machine manufacturing and electronic goods.	2.04
TCH	34°53′49.47″ N	108°56′42.44″ E	10	Coal and battery industry, aluminum electrolytic plant, and automobile industry.	0.79

**Table 2:** CoD for both road dust and construction dust among four selected cities.

	CD	XA	XY	BJ	TCH
RD					
XA			0.16	0.26	0.17
XY		0.23		0.34	0.21
BJ		0.24	0.2		0.22
TCH		0.39	0.2	0.31	

**Table 3:** Non-carcinogenic risk (HI) and carcinogenic risk (CR) of RD and CD PM_2.5_ in Guanzhong Plain.

		Non-Carcinogenic Risk								Carcinogenic Risk
Elements	Cities	Child				Adult				Adult
		HQ_ing_	HQ_inh_	HQ_dermal_	HI	HQ_ing_	HQ_inh_	HQ_dermal_	HI	CR_inh_
Co (in RD)	XA	2.79 × 10^−1^	2.73	9.74 × 10^−6^	3.01	2.99 × 10^−2^	4.17 × 10^−1^	5.49 × 10^−6^	4.47 × 10^−1^	1.50 × 10^−6^
	XY	1.87 × 10^−1^	1.83	6.52 × 10^−6^	2.02	2.00 × 10^−2^	2.80 × 10^−1^	3.68 × 10^−6^	3.00 × 10^−1^	1.01 × 10^−6^
	BJ	2.66 × 10^−1^	2.61	9.29 × 10^−6^	2.87	2.85 × 10^−2^	3.98 × 10^−1^	5.24 × 10^−6^	4.27 × 10^−1^	1.43 × 10^−6^
	TCH	1.07 × 10^−1^	1.05	3.73 × 10^−6^	1.15	1.14 × 10^−2^	1.60 × 10^−1^	2.10 × 10^−6^	1.71 × 10^−1^	5.76 × 10^−7^
Ni (in RD)	XA	1.85 × 10^−1^	5.03 × 10^−4^	1.92 × 10^−5^	1.86 × 10^−1^	1.98 × 10^−2^	7.69 × 10^−5^	1.08 × 10^−5^	1.99 × 10^−2^	1.33 × 10^−6^
	XY	2.18 × 10^−1^	5.92 × 10^−4^	2.25 × 10^−5^	2.18 × 10^−1^	2.33 × 10^−2^	9.04 × 10^−5^	1.27 × 10^−5^	2.34 × 10^−2^	1.56 × 10^−6^
	BJ	2.77 × 10^−1^	7.53 × 10^−4^	2.87 × 10^−5^	2.78 × 10^−1^	2.97 × 10^−2^	1.15 × 10^−4^	1.62 × 10^−5^	2.98 × 10^−2^	1.99 × 10^−6^
	TCH	1.23 × 10^−1^	3.33 × 10^−4^	1.27 × 10^−5^	1.23 × 10^−1^	1.31 × 10^−2^	5.09 × 10^−5^	7.16 × 10^−6^	1.32 × 10^−2^	8.81 × 10^−7^
Cu (in RD)	XA	2.14 × 10^−1^	5.95 × 10^−4^	1.99 × 10^−5^	2.14 × 10^−1^	2.29 × 10^−2^	9.09 × 10^−5^	1.12 × 10^−5^	2.30 × 10^−2^	
	XY	1.28 × 10^−1^	3.55 × 10^−4^	1.19 × 10^−5^	1.28 × 10^−1^	1.37 × 10^−2^	5.42 × 10^−5^	6.70 × 10^−6^	1.37 × 10^−2^	
	BJ	1.71 × 10^−1^	4.77 × 10^−4^	1.60 × 10^−5^	1.72 × 10^−1^	1.83 × 10^−2^	7.28 × 10^−5^	8.99 × 10^−6^	1.84 × 10^−2^	
	TCH	7.56 × 10^−2^	2.11 × 10^−4^	7.04 × 10^−6^	7.59 × 10^−2^	8.10 × 10^−3^	3.22 × 10^−5^	3.97 × 10^−6^	8.14 × 10^−3^	
As (in RD)	XA	3.63	1.01 × 10^−2^	7.44 × 10^−1^	4.39	3.89 × 10^−1^	1.55 × 10^−3^	1.14 × 10^−1^	5.05 × 10^−1^	7.04 × 10^−6^
	XY	2.44	6.80 × 10^−3^	4.99 × 10^−1^	2.94	2.61 × 10^−1^	1.04 × 10^−3^	7.63 × 10^−2^	3.38 × 10^−1^	4.72 × 10^−6^
	BJ	3.42	9.55 × 10^−3^	7.01 × 10^−1^	4.13	3.67 × 10^−1^	1.46 × 10^−3^	1.07 × 10^−1^	4.75 × 10^−1^	6.63 × 10^−6^
	TCH	2.31	6.44 × 10^−3^	4.73 × 10^−1^	2.79	2.47 × 10^−1^	9.83 × 10^−4^	7.21 × 10^−2^	3.20 × 10^−1^	4.47 × 10^−6^
Pb (in RD)	XA	2.47	6.89 × 10^−3^	4.61 × 10^−4^	2.48	2.65 × 10^−1^	1.05 × 10^−3^	2.60 × 10^−4^	2.66 × 10^−1^	1.56 × 10^−7^
	XY	1.70	4.74 × 10^−3^	3.17 × 10^−4^	1.71	1.83 × 10^−1^	7.24 × 10^−4^	1.79 × 10^−4^	1.83 × 10^−1^	1.07 × 10^−7^
	BJ	2.19	6.10 × 10^−3^	4.08 × 10^−4^	2.20	2.35 × 10^−1^	9.31 × 10^−4^	2.30 × 10^−4^	2.36 × 10^−1^	1.38 × 10^−7^
	TCH	1.14	3.17 × 10^−3^	2.12 × 10^−4^	1.14	1.22 × 10^−1^	4.84 × 10^−4^	1.20 × 10^−4^	1.23 × 10^−1^	7.15 × 10^−8^
Zn (in RD)	XA	1.32 × 10^−1^	3.69 × 10^−4^	1.84 × 10^−5^	1.32 × 10^−1^	1.41 × 10^−2^	5.64 × 10^−5^	1.04 × 10^−5^	1.42 × 10^−2^	
	XY	8.25 × 10^−2^	2.31 × 10^−4^	1.15 × 10^−5^	8.27 × 10^−2^	8.84 × 10^−3^	3.53 × 10^−5^	6.50 × 10^−6^	8.88 × 10^−3^	
	BJ	1.13 × 10^−1^	3.16 × 10^−4^	1.58 × 10^−5^	1.13 × 10^−1^	1.21 × 10^−2^	4.83 × 10^−5^	8.90 × 10^−6^	1.22 × 10^−2^	
	TCH	5.66 × 10^−2^	1.59 × 10^−4^	7.91 × 10^−6^	5.68 × 10^−2^	6.07 × 10^−3^	2.42 × 10^−5^	4.46 × 10^−6^	6.09 × 10^−3^	
Total (in RD)	XA	6.92	2.75	7.45 × 10^−1^	1.04 × 10	7.41 × 10^−1^	4.20 × 10^−1^	1.14 × 10^−1^	1.28	1.00 × 10^−5^
	XY	4.76	1.84	5.00 × 10^−1^	7.10	5.10 × 10^−1^	2.82 × 10^−1^	7.65 × 10^−2^	8.68 × 10^−1^	7.40 × 10^−6^
	BJ	6.44	2.63	7.02 × 10^−1^	9.77	6.90 × 10^−1^	4.01 × 10^−1^	1.07 × 10^−1^	1.20	1.02 × 10^−5^
	TCH	3.81	1.06	4.73 × 10^−1^	5.34	4.08 × 10^−1^	1.62 × 10^−1^	7.23 × 10^−2^	6.42 × 10^−1^	5.99 × 10^−6^
Co (in CD)	XA	1.51 × 10^−1^	1.48	5.27 × 10^−6^	1.63	1.62 × 10^−2^	2.26 × 10^−1^	2.97 × 10^−6^	2.42 × 10^−1^	8.13 × 10^−7^
	XY	1.60 × 10^−1^	1.57	5.58 × 10^−6^	1.73	1.71 × 10^−2^	2.39 × 10^−1^	3.15 × 10^−6^	2.56 × 10^−1^	8.60 × 10^−7^
	BJ	7.51 × 10^−2^	7.36 × 10^−1^	2.62 × 10^−6^	8.11 × 10^−1^	8.04 × 10^−3^	1.12 × 10^−1^	1.48 × 10^−6^	1.20 × 10^−1^	4.04 × 10^−7^
	TCH	1.19 × 10^−1^	1.17	4.16 × 10^−6^	1.29	1.28 × 10^−2^	1.79 × 10^−1^	2.35 × 10^−6^	1.91 × 10^−1^	6.42 × 10^−7^
Ni (in CD)	XA	1.42 × 10^−1^	3.85 × 10^−4^	1.47 × 10^−5^	1.42 × 10^−1^	1.52 × 10^−2^	5.88 × 10^−5^	8.27 × 10^−6^	1.53 × 10^−2^	1.02 × 10^−6^
	XY	1.27 × 10^−1^	3.44 × 10^−4^	1.31 × 10^−5^	1.27 × 10^−1^	1.36 × 10^−2^	5.25 × 10^−5^	7.39 × 10^−6^	1.36 × 10^−2^	9.09 × 10^−7^
	BJ	8.29 × 10^−2^	2.25 × 10^−4^	8.58 × 10^−6^	8.31 × 10^−2^	8.88 × 10^−3^	3.44 × 10^−5^	4.84 × 10^−6^	8.92 × 10^−3^	5.95 × 10^−7^
	TCH	1.24 × 10^−1^	3.37 × 10^−4^	1.28 × 10^−5^	1.24 × 10^−1^	1.33 × 10^−2^	5.15 × 10^−5^	7.24 × 10^−6^	1.34 × 10^−2^	8.91 × 10^−7^
Cu (in CD)	XA	9.00 × 10^−2^	2.51 × 10^−4^	8.38 × 10^−6^	9.02 × 10^−2^	9.64 × 10^−3^	3.83 × 10^−5^	4.73 × 10^−6^	9.68 × 10^−3^	
	XY	1.01 × 10^−1^	2.82 × 10^−4^	9.42 × 10^−6^	1.01 × 10^−1^	1.08 × 10^−2^	4.30 × 10^−5^	5.31 × 10^−6^	1.09 × 10^−2^	
	BJ	5.44 × 10^−2^	1.52 × 10^−4^	5.07 × 10^−6^	5.46 × 10^−2^	5.83 × 10^−3^	2.32 × 10^−5^	2.86 × 10^−6^	5.86 × 10^−3^	
	TCH	6.27 × 10^−2^	1.75 × 10^−4^	5.84 × 10^−6^	6.29 × 10^−2^	6.72 × 10^−3^	2.67 × 10^−5^	3.29 × 10^−6^	6.75 × 10^−3^	
As (in CD)	XA	2.76	7.69 × 10^−3^	5.65 × 10^−1^	3.33	2.95 × 10^−1^	1.17 × 10^−3^	8.62 × 10^−2^	3.83 × 10^−1^	5.34 × 10^−6^
	XY	2.92	8.14 × 10^−3^	5.97 × 10^−1^	3.52	3.12 × 10^−1^	1.24 × 10^−3^	9.12 × 10^−2^	4.05 × 10^−1^	5.65 × 10^−6^
	BJ	1.72	4.80 × 10^−3^	3.53 × 10^−1^	2.08	1.84 × 10^−1^	7.33 × 10^−4^	5.38 × 10^−2^	2.39 × 10^−1^	3.33 × 10^−6^
	TCH	2.95	8.23 × 10^−3^	6.04 × 10^−1^	3.56	3.16 × 10^−1^	1.26 × 10^−3^	9.23 × 10^−2^	4.10 × 10^−1^	5.71 × 10^−6^
Pb (in CD)	XA	1.30	3.61 × 10^−3^	2.42 × 10^−4^	1.30	1.39 × 10^−1^	5.51 × 10^−4^	1.36 × 10^−4^	1.40 × 10^−1^	8.15 × 10^−8^
	XY	1.29	3.58 × 10^−3^	2.40 × 10^−4^	1.29	1.38 × 10^−1^	5.47 × 10^−4^	1.35 × 10^−4^	1.39 × 10^−1^	8.09 × 10^−8^
	BJ	7.47 × 10^−1^	2.08 × 10^−3^	1.39 × 10^−4^	7.49 × 10^−1^	8.00 × 10^−2^	3.17 × 10^−4^	7.84 × 10^−5^	8.04 × 10^−2^	4.69 × 10^−8^
	TCH	7.87 × 10^−1^	2.19 × 10^−3^	1.47 × 10^−4^	7.90 × 10^−1^	8.44 × 10^−2^	3.35 × 10^−4^	8.27 × 10^−5^	8.48 × 10^−2^	4.95 × 10^−8^
Zn (in CD)	XA	6.70 × 10^−2^	1.88 × 10^−4^	9.36 × 10^−6^	6.72 × 10^−2^	7.18 × 10^−3^	2.86 × 10^−5^	5.28 × 10^−6^	7.21 × 10^−3^	
	XY	6.13 × 10^−2^	1.72 × 10^−4^	8.56 × 10^−6^	6.14 × 10^−2^	6.56 × 10^−3^	2.62 × 10^−5^	4.83 × 10^−6^	6.60 × 10^−3^	
	BJ	3.07 × 10^−2^	8.60 × 10^−5^	4.29 × 10^−6^	3.08 × 10^−2^	3.29 × 10^−3^	1.31 × 10^−5^	2.42 × 10^−6^	3.31 × 10^−3^	
	TCH	4.32 × 10^−2^	1.21 × 10^−4^	6.04 × 10^−6^	4.34 × 10^−2^	4.63 × 10^−3^	1.85 × 10^−5^	3.41 × 10^−6^	4.65 × 10^−3^	
Total (in CD)	XA	4.50	1.49	5.65 × 10^−1^	6.56	4.82 × 10^−1^	2.28 × 10^−1^	8.64 × 10^−2^	7.97 × 10^−1^	7.25 × 10^−6^
	XY	4.65	1.58	5.98 × 10^−1^	6.83	4.98 × 10^−1^	2.41 × 10^−1^	9.14 × 10^−2^	8.31 × 10^−1^	7.50 × 10^−6^
	BJ	2.71	7.43 × 10^−1^	3.53 × 10^−1^	3.81	2.90 × 10^−1^	1.14 × 10^−1^	5.39 × 10^−2^	4.58 × 10^−1^	4.38 × 10^−6^
	TCH	4.09	1.18	6.05 × 10^−1^	5.87	4.38 × 10^−1^	1.80 × 10^−1^	9.24 × 10^−2^	7.10 × 10^−1^	7.30 × 10^−6^

**Table 4:** LUR model of risk-related variable.

Variable	Equation	R^2^	Sig.
HI (THM)	5.805 + 0.497 × TransStation (100) + 0.002 × TransStation (2000)	0.144	0.024
HI (Co)	1.274 + 0.142 × TransStation (100) + 0.003 × Restaurant (300) + 0.092 × 1stRoad (300) + 0.001 × TransStation (1500)	0.185	0.047
HI (Pb)	1.062 + 0.128 × TransStation (100) + 0.018 × LifeService (100)+0.069 × 1stRoad (300) + 0.0005 × Enterprise (1500)	0.230	0.015

## Data Availability

The observational data obtained in this study are available from the corresponding authors upon request (zhangqian2018@xauat.edu.cn).

## Supplementary Materials

The following supporting information can be downloaded at: https://www.mdpi.com/article/10.3390/toxics11080676/s1, Figure S1: the specific location of sampling sites; Table S1: Values of parameters for health risk calculation; Table S2: Summary of reference doses (RfD) and slope factors (SF) of heavy metals; Table S3: List of major variables for LUR model; Table S4a: Element concentration of road dust PM_2.5_ in four selected cities; Table S4b: Element concentration of construction dust PM_2.5_ in four selected cities; Table S5a: Water-soluble ions of road dust PM_2.5_ in four selected cities; Table S5b: Water-soluble ions of construction dust PM_2.5_ in four selected cities; Table S6a: Diagnostic ratio of water-soluble ions in Guanzhong Plain Table S7a: Carbonaceous frictions of road dust PM_2.5_ in four selected cities; Table S7b: Carbonaceous frictions of construction dust PM_2.5_ in four selected cities; Table S8: Detail information of each LUR model.
