# Supplementary material for: Chemical Source Profiles and Toxicity Assessment of Urban Fugitive Dust PM2.5 in Guanzhong Plain, China
Source: Toxics. 2023 Aug 7;11(8):676. doi: 10.3390/toxics11080676 (PMC10458601; doi:10.3390/toxics11080676)
Supplement: Supplementary file 1 [file toxics-11-00676-s001.zip › toxics-2545499-supplementary.pdf]

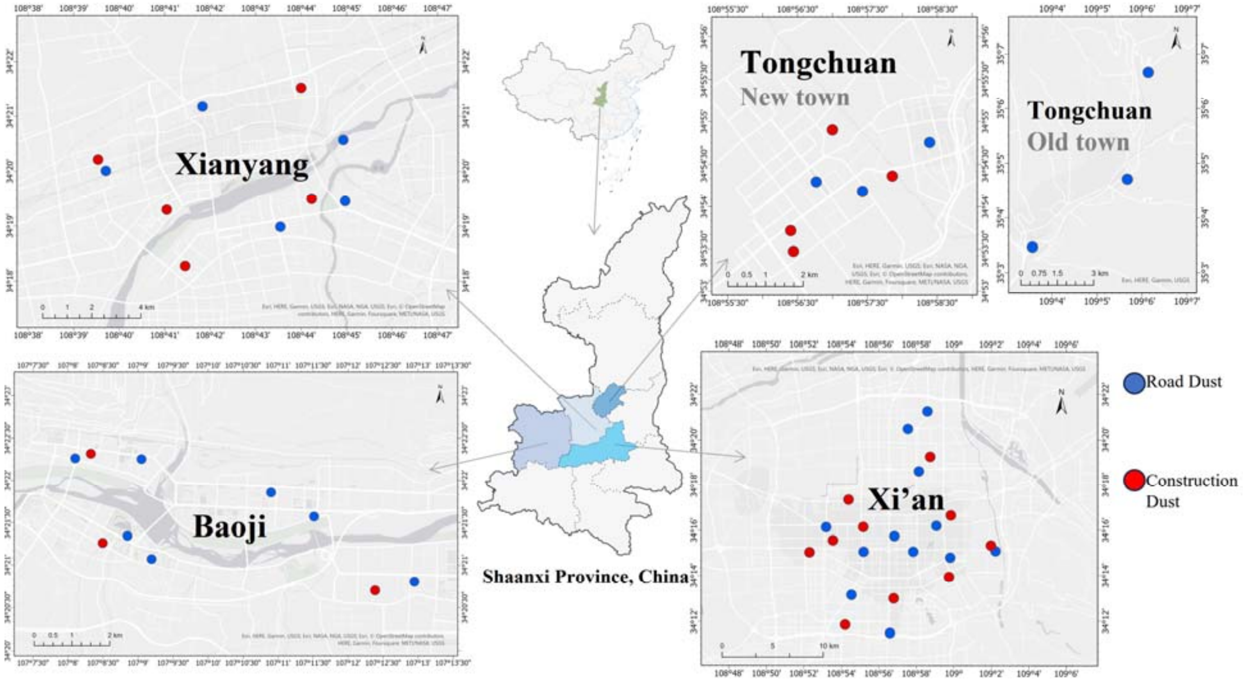

Figure S1. The specific loaction of sampling sites.

Table S1. Values of parameters for health risk calculation.

Indicators	Parameters	Defination	Units	Adult	Children	Reference
Exposure factors	EF	Exposure frequency	day/year	350	350	[1]
	ED	Exposure duration	year	24	6	[1]
	BW	Body weight	kg	70	15	[2]
	AT	Average exposed time(non-carcinogen)	day	ED×365	ED×365	[1]
		Average exposed time(carcinogen)	day	70×365	70×365	[1]
	CF	Conversion factor	-	10^{-6}	10^{-6}	
	C	Concentration of heavy metals	mg/kg	-	-	this study
Ingestion	ingR	Ingestion rate of dust	mg/day	100	200	[1]
Inhalation	inhR	Inhalation rate of dust	m^3/day	20	7.6	[3]
Dermal contact	PEF	Particle emission factor	m^3/kg	1.36×10^9	1.36×10^9	[1]
	SL	Skin adherence fatcor	mg/cm^2	0.07	0.2	[1]
	SA	Exposed skin area	cm^2	5700	2800	[1]
	ABS	Dermal absorption factor (except As)	-	0.001	0.001	[1]
		Dermal absorption factor (for As)	-	0.03	0.03	[1]

Table S2. Summary of reference doses (RfD) and slope factors (SF) of heavy metals.

	Mn	Co	Ni	Cu	As	Pb	Zn
RfD_{ing}(mg/kg/day)	4.60E-02	2.00E-02	2.00E-02	4.00E-02	3.00E-04	3.50E-03	3.00E-01
RfD_{inh}(mg/kg/day)	1.43E-05	5.71E-06	2.06E-02	4.02E-02	3.01E-04	3.52E-03	3.00E-01
RfD_{dermal}(mg/kg/day)	1.84E-03	1.60E-02	5.40E-03	1.20E-02	1.23E-04	5.25E-04	6.00E-02
Sf_{inh}(mg/kg/day)		6.30E-01	8.40E-01		1.51E+01	4.20E-02	

Reference: [4,5]

Table S3. List of major variables for LUR model.

Factors	Independent variables	Descriptions	buffer radii	unit
Traffic related	Transport Station (TransStation)	number of bus stops, metro stations and train stations		count
	automobile related (AutoRelated)	number of dealerships, garages, and gas stations		count
	first-class road (1stRaod)	length of road	100m	km
	second-class road (2ndRaod)	length of road	300m	km
	third-class road (3rdRaod)	length of road	500m	km
Domestic related	Restaruant	number of restaurants	1000m	count
	Hotel	number of hotels	1500m	count
	Residence	number of residential buildings	2000m	count
	LifeService	number of express points, barber shops, toilet and so on		count
Other	Enterprise	number of enterprises and companies		count

Table S4a. Element concentration of road dust $PM_{2.5}$ in four selected cities.

	XA		XY		BJ		TCH	
	Concentration, μg/g		Concentration, μg/g		Concentration, μg/g		Concentration, μg/g	
	MEAN	SD	MEAN	SD	MEAN	SD	MEAN	SD
K	45132	12828	33069	6762	35151	5340	26976	2170
Ca	339562	91921	262562	34604	262806	50716	225891	8967
Fe	49705	8092	45797	6733	51502	5746	37885	2414
Mn	1457	390	1127	229	1195	143	837	60
Ti	3983	792	3913	432	3938	335	4336	1295
V	110	46	90	23	101	27	54	16
Sc	3245	1354	1958	409	2307	625	1441	98
Co	490	277	292	101	416	159	167	34
Ni	313	136	341	147	433	139	192	32
Cu	724	264	399	101	536	157	237	25
As	83	38	57	19	80	33	54	19
Se	32	17	19	7	27	10	11	3
Br	55	35	21	5	48	28	21	5
Cl	7181	3173	4385	1267	5462	1792	2584	558
Sr	1249	448	1354	470	1117	388	661	74
Ba	2007	841	938	372	1179	306	666	116
Pb	743	342	466	134	599	151	311	71
Ga	195	105	120	38	161	64	76	22
Zn	3318	1310	1935	496	2651	371	1328	183
Total	437499	46730	358843	50216	369711	64231	303730	9850

Table S4b. Element concentration of construction dust $PM_{2.5}$ in four selected cities.

	XA		XY		BJ		TCH	
	Concentration, μg/g		Concentration, μg/g		Concentration, μg/g		Concentration, μg/g	
	MEAN	SD	MEAN	SD	MEAN	SD	MEAN	SD
K	32364	8691	34613	6046	21856	4981	32512	3897
Ca	228523	40349	263556	41106	232551	11358	240964	17035
Fe	47721	10234	50996	6811	37719	12696	42039	6828
Mn	1213	273	1276	180	909	183	1029	110
Ti	3481	777	3304	551	2729	396	3375	459
V	80	34	83	13	63	12	73	22
Sc	1647	529	2164	430	1176	322	1491	295
Co	236	124	250	60	117	59	186	61
Ni	222	98	198	30	130	32	194	66
Cu	282	106	317	71	170	20	196	52
As	65	21	68	14	40	5	69	23
Se	15	9	17	4	8	3	12	4
Br	27	19	29	9	14	8	19	12
Cl	3815	1795	3885	832	1766	512	2540	792
Sr	848	273	1000	127	650	90	862	271
Ba	950	522	826	230	495	162	671	327
Pb	355	119	352	116	204	36	216	35
Ga	102	43	106	17	56	18	79	14
Zn	1572	679	1438	268	721	130	1015	157
Total	323517	46730	364477	49025	301375	9954	327540	22600

Table S5a. Water-soluble ions of road dust $PM_{2.5}$ in four selected cities.

	XA		XY		BJ		TCH	
	Concentration, μg/g		Concentration, μg/g		Concentration, μg/g		Concentration, μg/g	
	MEAN	SD	MEAN	SD	MEAN	SD	MEAN	SD
F^-	16800	9188	9953	3292	9545	3193	5003	539
Cl^-	19667	9799	12604	4031	15147	5890	6119	1387
NO_3^-	6901	4020	2944	1269	480	146	2268	863
SO_4^{2-}	39025	17480	19065	5428	17517	6201	10955	3179
Na^+	48226	22754	54980	22391	44159	23686	19614	4170
NH_4^+	2563	2250	3809	2139	3261	2064	2951	2172
K^+	4161	1557	1865	392	2388	1225	2523	591
Mg^{2+}	10625	3925	12036	4921	11993	4381	5318	397
Ca^{2+}	156775	36490	197482	32254	158356	50294	133626	29401
Total	285334	78046	312979	70592	257481	86247	186560	29670

Table S5b. Water-soluble ions of construction dust $PM_{2.5}$ in four selected cities.

	XA		XY		BJ		TCH	
	Concentration, μg/g		Concentration, μg/g		Concentration, μg/g		Concentration, μg/g	
	MEAN	SD	MEAN	SD	MEAN	SD	MEAN	SD
F^-	9953	3292	12343	8533	3783	1340	6282	1920
Cl^-	12604	4031	14393	10645	3530	1569	7235	2419
NO_3^-	2944	1269	6063	6307	1648	499	4255	1339
SO_4^{2-}	19065	5428	27213	18688	9859	1771	10744	3904
Na^+	54980	22391	34461	12739	10529	3216	28589	10130
NH_4^+	3809	2139	1900	824	1637	1221	2848	2310
K^+	1865	392	3164	747	3156	1233	5048	2986
Mg^{2+}	12036	4921	10236	4611	3254	1259	8682	1125
Ca^{2+}	197482	32254	176612	41472	115077	34678	176266	12744
Total	182196	57915	284412	98653	150960	43101	24983	10040

Table S6. Diagnostic ratio of water-soluble ions in Guanzhong Plain.

Location	Sample type	Sampling time	K^+/K	Ca^{2+}/Ca	NO_3^-/SO_4^{2-}	SO_4^{2-}/Ca^{2+}	SO_4^{2-}/OC	SO_4^{2-}/EC	SO_4^{2-}/K^+	As/Fe
XA	RD	2019.6-2019.11	0.09	0.46	0.18	0.25	0.19	2.30	9.38	0.002
	CD		0.09	0.55	0.27	0.11	0.20	4.11	10.22	0.001
XY	RD		0.06	0.75	0.15	0.10	0.13	1.42	10.22	0.001
	CD		0.09	0.67	0.22	0.15	0.22	10.16	8.60	0.001
BJ	RD		0.07	0.60	0.03	0.11	0.10	1.02	7.33	0.002
	CD		0.14	0.49	0.17	0.09	0.06	2.25	3.12	0.001
TCH	RD		0.09	0.59	0.21	0.08	0.08	0.55	4.34	0.001
	CD		0.16	0.73	0.40	0.06	0.12	5.37	2.13	0.002
Guanzhong Plain	RD		0.08	0.60	0.14	0.13	0.13	1.28	7.82	0.002
	CD		0.12	0.61	0.26	0.10	0.13	4.45	4.62	0.001

Table S7a. Carbonaceous frictions of road dust $PM_{2.5}$ in four selected cities.

	XA		XY		BJ		TCH	
	Concentration, μg/g		Concentration, μg/g		Concentration, μg/g		Concentration, μg/g	
	MEAN	SD	MEAN	SD	MEAN	SD	MEAN	SD
TC	217791	72545	164887	27073	190454	18216	150421	11183
OC	200843	66567	151478	24239	173282	17564	130344	11432
EC	16948	7486	13409	3534	17172	4359	20077	3364
OC/EC	11.85	8.89	11.30	6.86	10.09	4.03	6.49	3.40
OC1	11943	5617	6729	2274	9971	3284	4692	903
OC2	30451	12691	18902	5679	25025	4974	12879	1901
OC3	94176	38215	61796	10502	76835	10863	51870	6777
OC4	61855	11682	62841	8040	60550	3051	59226	4437
EC1	17700	8026	12505	3305	16415	3570	19627	3102
EC2	1648	829	2115	612	1657	742	2081	446
EC3	106	44	0	0	0	0	89	9
OP	1926	1257	1211	657	901	686	1676	707

Table S7b. Carbonaceous frictions of construction dust $PM_{2.5}$ in four selected cities.

	XA		XY		BJ		TCH	
	Concentration, μg/g		Concentration, μg/g		Concentration, μg/g		Concentration, μg/g	
	MEAN	SD	MEAN	SD	MEAN	SD	MEAN	SD
TC	102254	72545	128133	32922	161430	78778	90170	6991
OC	97619	66567	125455	32491	157043	77512	88171	6035
EC	4635	7486	2678	2408	4387	2254	1999	1098
OC/EC	21.06	8.89	46.84	13.49	35.80	34.39	44.10	5.50
OC1	5702	5617	7219	2800	6506	3011	4078	1370
OC2	11802	12691	21362	13115	13027	6208	7866	2231
OC3	35333	38215	46235	17193	40645	20623	25391	4500
OC4	42611	11682	45129	6573	95103	49679	47306	2725
EC1	4927	8026	6872	5591	3169	2217	4096	1056
EC2	1845	829	1317	310	2753	1407	1433	299
EC3	206	32	0	0	226	354	0	0
OP	2171	2344	5510	6959	1761	2427	3530	671

Table S8. Detail information of each LUR model.

Variables	Factors	Coefficient (B)	Standardized Coefficient (β)	VIF
HI(THM)	constant	5.805		
	TransStation (100)	0.497	0.292	1.352
	TransStation (2000)	0.002	0.135	1.352
HI(Co)	constant	1.274		
	TransStation (100)	0.142	0.234	1.518
	Restaruant (300)	0.003	0.092	1.683
	1stRoad (300)	0.092	0.119	1.358
	TransStation (1500)	0.001	0.111	1.697
HI(Pb)	constant	1.062		
	TransStation (100)	0.128	0.304	1.333
	LifeService (100)	0.018	0.111	1.505
	1stRoad (300)	0.069	0.128	1.405
	Enterprise (1500)	0.0005	0.081	1.394

References:

1. USEPA. *Supplemental Guidance for Developing Soil Screening Levels for Superfund Sites*; Office of Solid Waste and Emergency Response, US Environmental Protection Agency., Washington,D.C., 2001. URL: https://semspub.epa.gov/work/HQ/175878.pdf
2. USEPA. Human Health Evaluation Manual. In *Risk Assessment Guidance for Superfund*; Office of emergency and remedial response, US Environmental Protection Agency, Washington,D.C., 1989; Volume 1, p. 179. URL: https://www.epa.gov/sites/default/files/2015-09/documents/rags_a.pdf
3. Van den Berg, R. *Human Exposure to Soil Contamination: A Qualitative and Quan titative Analysis towards Proposals for Human Toxicological Intervention Values.*; National Institute of Public Health and Environmental Protection (RIVM): Bilthoven, The Netherlands, 1995. URL: https://www.rivm.nl/bibliotheek/rapporten/725201011.pdf
4. Safiur Rahman, M.; Khan, M.D.H.; Jolly, Y.N.; Kabir, J.; Akter, S.; Salam, A. Assessing risk to human health for heavy metal contamination through street dust in the Southeast Asian Megacity: Dhaka, Bangladesh. *Science of The Total Environment* **2019**, *660*, 1610-1622, doi:https://doi.org/10.1016/j.scitotenv.2018.12.425.
5. Heidari, M.; Darijani, T.; Alipour, V. Heavy metal pollution of road dust in a city and its highly polluted suburb; quantitative source apportionment and source-specific ecological and health risk assessment. *Chemosphere* **2021**, *273*, 129656, doi:https://doi.org/10.1016/j.chemosphere.2021.129656.
